# Supplementary material for: Anti-Inflammatory Effects of Analogues of N-Acyl Homoserine Lactones on Eukaryotic Cells
Source: Int J Mol Sci. 2020 Dec 11;21(24):9448. doi: 10.3390/ijms21249448 (PMC7764250; doi:10.3390/ijms21249448)
Supplement: Supplementary file 1 [file ijms-21-09448-s001.pdf]

Supplementary File

Anti-Inflammatory Effects of *N*-Acyl Homoserine Lactones Analogues on Eukaryotic Cells

Agathe Peyrottes [1,2], Garance Coquant [2], Loïc Brot [2], Dominique Rainteau [2], Philippe Seksik [2,3], Jean-Pierre Grill [2] and Jean-Maurice Mallet [1]

[1] Laboratoire des Biomolécules (LBM), Département de chimie, École Normale Supérieure, PSL University, Sorbonne Université, CNRS, Paris, France.

[2] Sorbonne Université, INSERM, Centre de recherche Saint-Antoine, APHP, Hôpital Saint-Antoine, Microbiote Intestin et Inflammation, Paris, France.

[3] Service de gastroentérologie et nutrition, Hôpital Saint-Antoine, APHP, Paris, France.

* Correspondence: philippe.seksik@sat.aphp.fr; Tel.: + 33(0)1.49.28.31.62 , Fax : + 33(0)1.49.28.31.88

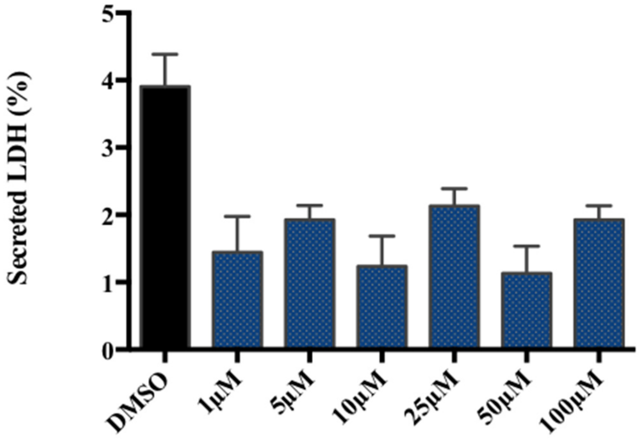

Supplementary S1: Cytotoxicity of 3-oxo-C12-HSL treatment on Caco-2/TC7 cells in stimulated state as measured by LDH release. Cells were treated with increasing doses of 3-oxo-C12-HSL combined to IL-1ß. The points are the mean value of different replicates (n ≥ 3) ± SEM. No statistical difference was observed between conditions.

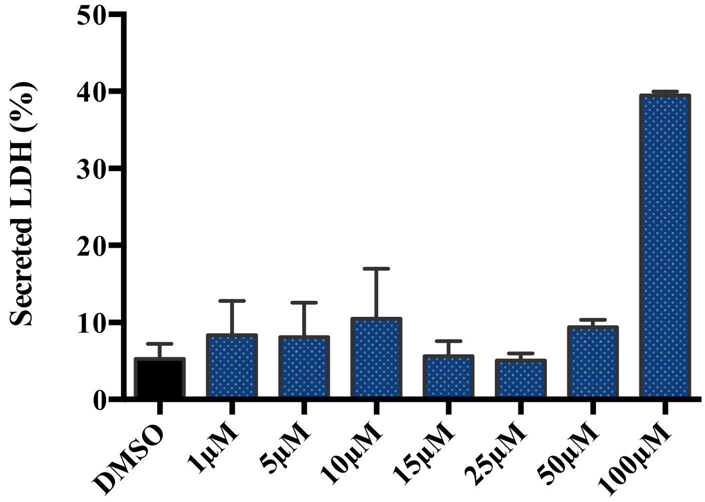

Supplementary S2: Cytotoxicity of 3-oxo-C12-HSL treatment on RAW264.7 cells in stimulated state as measured by LDH release. Cells were treated with increasing doses of 3-oxo-C12-HSL combined to LPS/TNF-α. The points are the mean value of different replicates (n = 3) ± SEM. No statistical difference was observed between conditions.

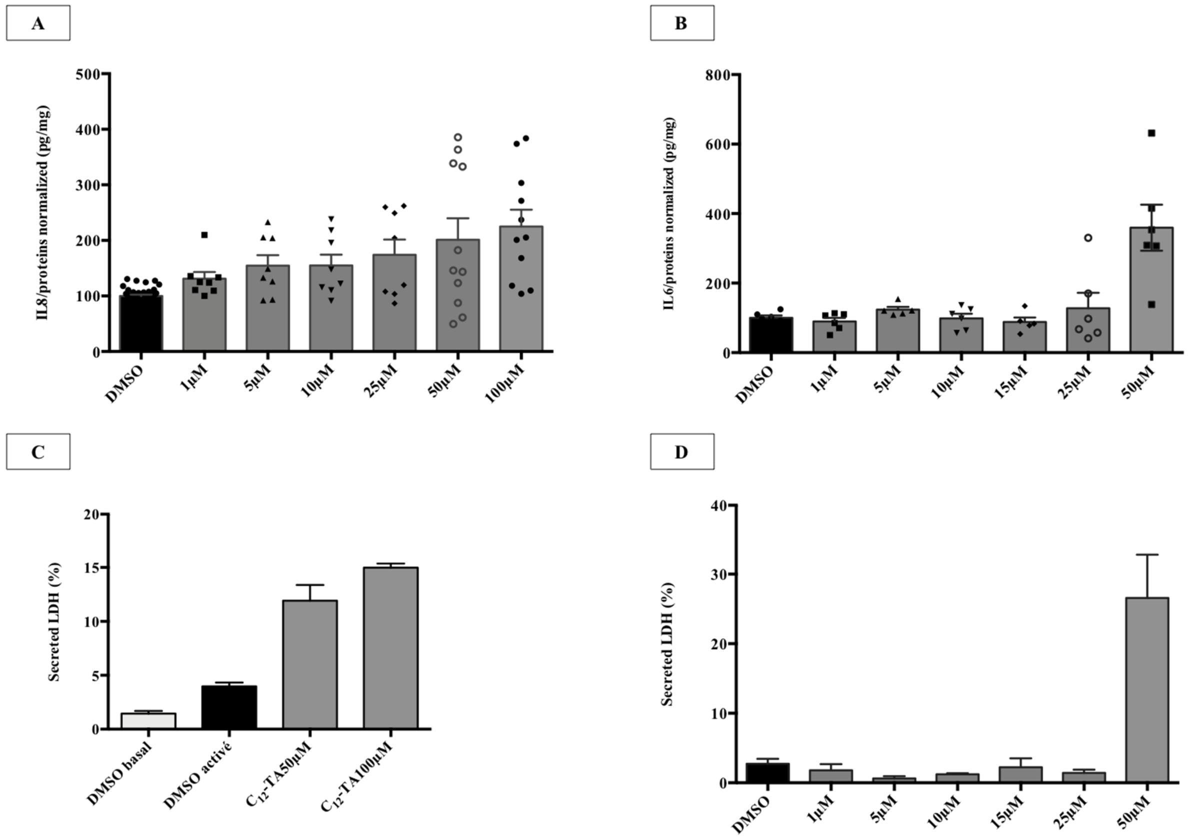

Supplementary S3: Compared biological effects of tetramic acid (3) on cell lines Caco-2/TC7 and RAW264.7. **A** (resp. **B**) : IL-8 response of Caco-2/TC7 cells (resp. IL-6 response of Raw 264.7 cells) to stimulation in presence of increasing doses of tetramic acid (3). **C** (resp. **D**): secreted LDH in Caco-2/TC7 cells (resp. RAW264.7 cells) to stimulation in presence of increasing doses of tetramic acid (3). The points are the mean value of different replicates (n ≥ 3) ± SEM.

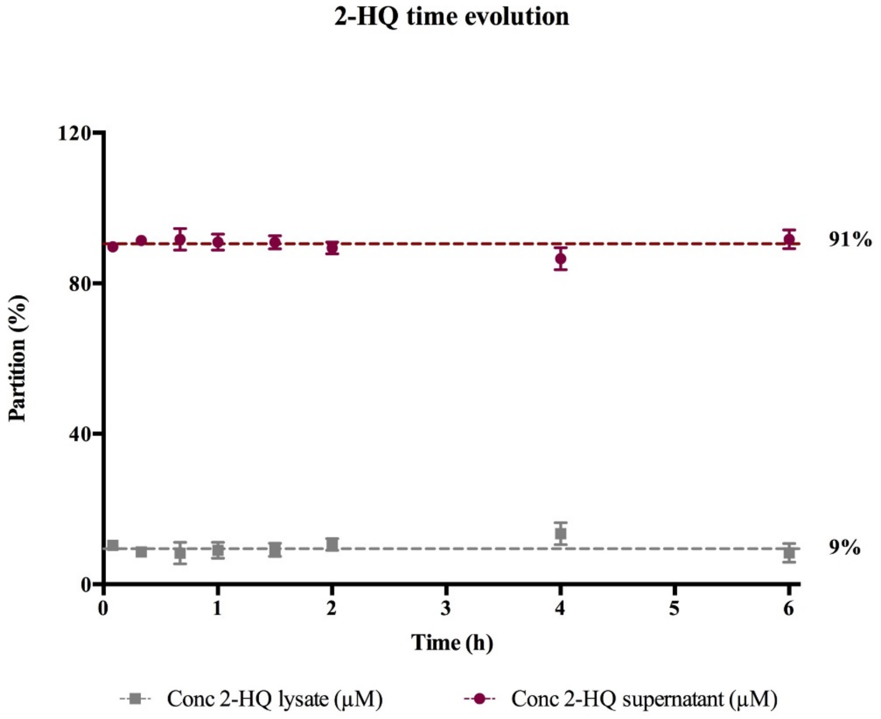

Supplementary S4: 2-HQ distribution over time

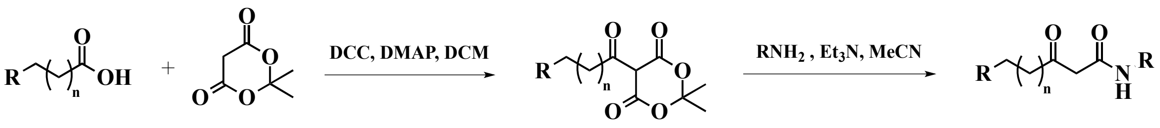

Supplementary S5: Historic AHL synthetic method.

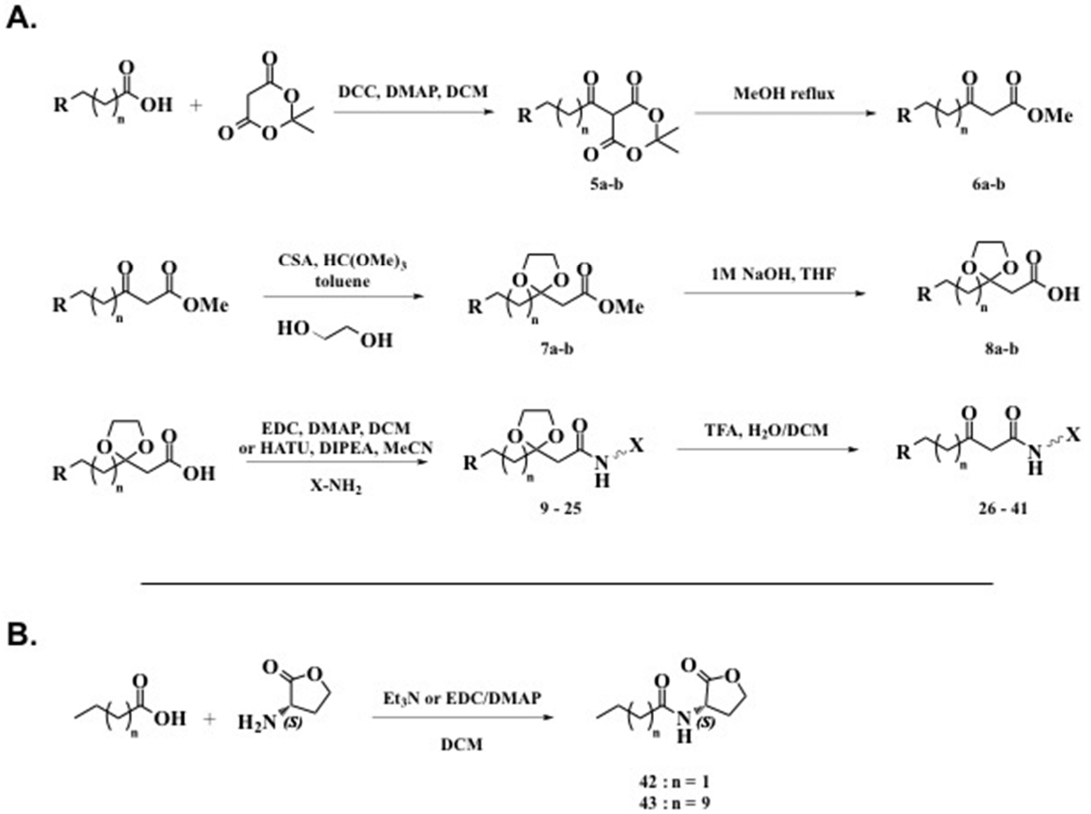

Supplementary S6: Improved total synthesis of natural AHLs and their analogues. **A**: synthetic path to access C3-keto-AHL (n = 1-7, R =CH_3 or N_3). **B**: synthetic path to access un-C3-substituted AHLs.

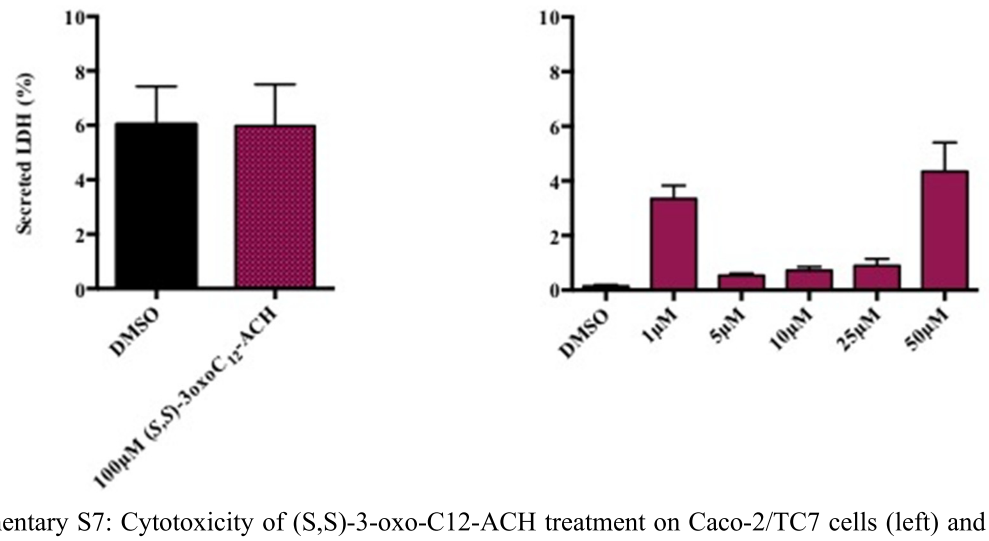

Supplementary S7: Cytotoxicity of (S,S)-3-oxo-C12-ACH treatment on Caco-2/TC7 cells (left) and RAW264.7 macrophages (right)in stimulated state as measured by LDH release. Cells were treated with increasing doses of (S,S)-3-oxo-C12-ACH. The points are the mean value of different replicates (n ≥ 3) ± SEM. No statistical difference was observed between conditions.

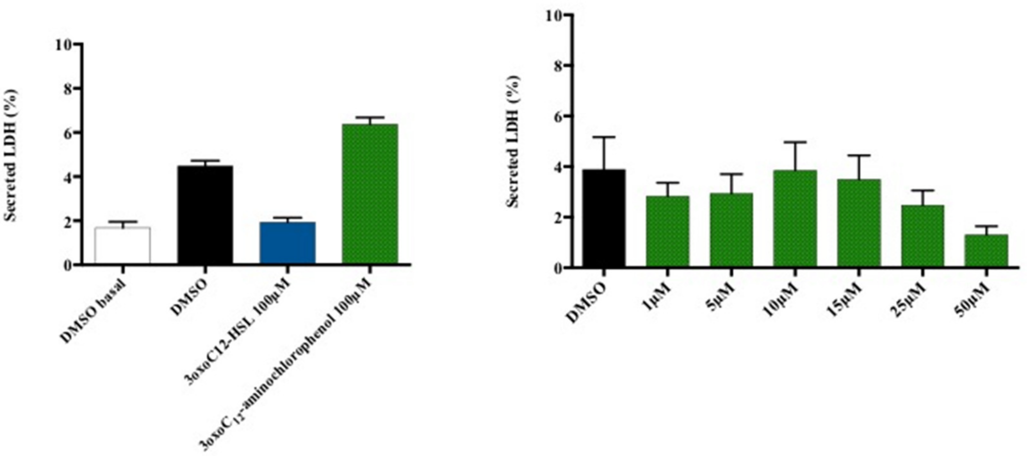

Supplementary S8: Cytotoxicity of 3-oxo-C12-2,4-aminochlorophenol treatment on Caco-2/TC7 cells (left) and RAW264.7 macrophages (right)in stimulated state as measured by LDH release. Cells were treated with increasing doses of 3-oxo-C12-2,4-aminochlorophenol. The points are the mean value of different replicates (n ≥ 3) ± SEM. No statistical difference was observed between conditions.
